# Hypoxic preconditioning improves long‐term functional outcomes after neonatal hypoxia–ischemic injury by restoring white matter integrity and brain development

**DOI:** 10.1111/cns.13102

**Published:** 2019-01-28

**Authors:** Ming‐Yue Xu, Yang‐Fan Wang, Peng‐Ju Wei, Yan‐Qin Gao, Wen‐Ting Zhang

**Affiliations:** ^1^ State Key Laboratory of Medical Neurobiology, Institutes of Brain Science, Collaborative Innovation Center for Brain Science Fudan University Shanghai China

**Keywords:** development, hypoxic preconditioning, hypoxic/ischemic, inflammation, white matter injury

## Abstract

**Aims:**

Neonatal hypoxia–ischemia (H/I) results in gray and white matter injury, characterized by neuronal loss, failure of neural network formation, retarded myelin formation, and abnormal accumulation of oligodendrocyte progenitor cells (OPCs). These changes lead to severe neurological deficits and mortality. Sublethal hypoxic preconditioning (HPC) can protect the developing brain against H/I. However, limited evidence is available concerning its effect on white matter injury.

**Methods:**

In this study, P6 neonatal Sprague‐Dawley rats were subjected to normoxic (21% O_2_) or HPC (7.8% O_2_) for 3 hours followed 24 hours later by H/I brain injury. Neurological deficits were assessed by gait, righting reflex, foot fault, and Morris water maze tests. Compound action potential of the corpus callosum was recorded 35 days after surgery, and the correlation between axon myelination and neurological function was determined.

**Results:**

Hypoxic preconditioning significantly attenuated H/I brain injury at 7 days and remarkably improved both sensorimotor and cognitive functional performances up to 35 days after H/I. HPC‐afforded improvement in long‐term neurological outcomes was attributable, at least in part, to restoration of the differentiation and maturation capacity in oligodendrocyte progenitor cells, amelioration of microglia/macrophage activation and neuroinflammation, and continuation of brain development after H/I.

**Conclusions:**

Hypoxic preconditioning restores white matter repair, development, and functional integrity in developing brain after H/I brain injury.

## 1 INTRODUCTION

Perinatal hypoxic/ischemic (H/I) brain injury, induced by insufficient supply of oxygen and glucose to the brain,^1^ occurs in 3 per 1000 preterm infants (<36 weeks of gestation). H/I brain injury is one of the major causes of mortality and morbidity in newborns.^2^ Due to extensive neuronal and glial loss, and failure of neural circuitry development in immature brains, most survivors suffer from lifelong neurodevelopmental impairments.^2^, ^3^ White matter is particularly susceptible to H/I brain injury. White matter injury, including disruption of myelin formation, axonal damage, and oligodendroglia loss,^4^, ^5^, ^6^ has proven to be the leading cause of cerebral palsy, mental retardation, and neurobehavioral disabilities in survivors.^7^, ^8^ Among oligodendrocyte linage, late oligodendrocyte progenitors have been reported to be selectively more vulnerable to H/I injury than early oligodendrocyte progenitors and immature oligodendrocytes.^9^, ^10^, ^11^ Although immature neurons seem to be more resistant to transient H/I‐induced cell death,^8^, ^12^ white matter necrosis, characterized by progressive degeneration of premature oligodendrocytes and axons, results in retrograde axonal degeneration. This in turn causes secondary neuronal loss in cortical and subcortical gray matter following H/I brain injury.^3^, ^13^ Thus, to minimize neurodevelopmental impairments, it is critical that the survival and maturation of late oligodendrocyte progenitors be maintained to facilitate the development of myelin and to rebuild new circuit connections to restore axonal conductive sensitivity in neonates after H/I brain injury.^14^

Microglia, resident macrophages of the central nervous system, play a fundamental role in the development of the brain.^15^ Indeed, microglia regulate the proliferation and differentiation of neurons and oligodendrocytes,^16^, ^17^ promote neurite development and regeneration,^18^ modulate synapse pruning and remodeling,^19^ and clear debris of normal apoptotic cells.^20^ Microglia activation has been increasingly recognized as a major contributor to pathophysiological outcomes in the developing brain.^21^, ^22^ Thus, therapeutic strategies restricting microglia activation and production of pro‐inflammatory cytokines may be beneficial to the survival and maturation of neurons and oligodendroglia.^23^, ^24^, ^25^, ^26^

Sublethal hypoxic preconditioning (HPC) enhances the tolerance of cells, tissues, and even organism to subsequent lethal insults like ischemia or hypoxia in neonates and adults.^27^, ^28^ Previous research demonstrated that HPC protected the developing brain against H/I injury by attenuating neuronal death,^29^ reducing microglia activation,^30^ and enhancing neurogenesis.^31^ Inducible expression of the transcriptional factor hypoxia inducible factor‐1 (HIF‐1) seems to be essential for HPC‐mediated neuroprotection, as knockout of HIF‐1α eliminates the protective effect of HPC.^32^ Furthermore, HPC increases glycogen levels to delay energy depletion^33^ and downregulates cerebral metabolic demand and energy‐consuming processes by suppressing ATPase activity and protein synthesis after cerebral ischemia.^27^, ^34^ Although HPC has been reported to reduce acute myelin loss caused by neonatal H/I injury,^35^ the long‐term effects of HPC on white matter integrity are still unknown.

In this study, we hypothesized that HPC could reactivate normal development of the postnatal brain parenchyma to promote long‐term neurological recovery after H/I injury. To test this hypothesis, the impact of HPC on long‐term neurological outcomes and brain parenchyma integrity after white matter injury was assessed in a well‐established neonatal hypoxic–ischemic brain injury model. Our results suggest that HPC promotes long‐term functional recovery of sensorimotor and cognitive deficits by partially rehabilitating the abnormal development of brain parenchyma by revitalizing oligodendrocyte progenitor cell (OPC) maturation and suppressing excessive microglia activation.

## 2 METHODS

### 2.1 Hypoxic preconditioning and model of neonatal H/I brain injury

Pregnant Sprague‐Dawley rats (Laboratory Animal Center, Chinese Academy of Science, Shanghai, China) were housed in a 12‐hour light–dark cycle with free access to food and water. All procedures were approved by the Animal Care and Use Committee at Fudan University. Animals were randomly assigned to H/I, HPC + H/I or sham groups. Six‐day‐old pups were treated with HPC in a chamber balanced with 7.8% O_2_/92.2% N_2_ and submersed in a 37°C water bath to maintain normothermia for 3 hours. Sham animals were exposed to ambient air (21% O_2_) at 37°C for the same length of time. Twenty‐four hours later, neonates were subjected to sham or H/I brain surgery as previously described.^36^ Briefly, seven‐day‐old pups were anesthetized with 1.5% isoflurane mixed with ambient air, and the left common carotid artery was ligated permanently in all pups except those in the sham group. After a 1.5‐hour recovery period, pups were again exposed to 7.8% O_2_/92.2% N_2_ (H/I and HPC + H/I groups) or ambient air (sham group) for 2.5 hours.

### 2.2 Neurological function evaluation

Tests assessing gait and righting reflex were performed daily as previously described^37^ for 5 days after H/I. Long‐term neurological deficits were assessed by foot fault and Morris water maze tests up to 35 days after H/I as previously described.^38^ All tests were performed by investigators blinded to experimental group assignment.

### 2.3 Immunofluorescence staining

Coronal brain sections (25 μm thick) were used for immunohistochemistry. Sections were washed three times in phosphate‐buffered saline (PBS) solution, blocked with 10% goat serum for 1 hour, followed by 1‐hour incubation in a primary antibody (listed in supplementary file, Table S2) at 37°C and overnight at 4°C. The following day, brain sections were washed three times in PBS containing 0.3% Triton‐X 100 (PBST) and then incubated with a species‐specific secondary antibody conjugated with DyLight 488 or DyLight 594 (Jackson ImmunoResearch Laboratories, West Grove, PA). Sections were then mounted with Fluoromount‐G (Southern Biotech, Birmingham, AL). Images were captured using confocal microscopy (Olympus America, Center Valley, PA).

### 2.4 Examination of newly proliferated cells

5‐Bromo‐2′‐deoxyuridine (BrdU) (Sigma‐Aldrich, St. Louis, MO), an S‐phase marker, was used to label newly proliferated cells. BrdU was intraperitoneally injected twice per day at 50 mg/kg body weight from days 3 to 7 after H/I injury. Coronal brain sections were prepared at 7 or 14 days after H/I injury. Sections were incubated in 2N HCl at 37°C for 1 hour, followed by neutralization in 0.1 mol/L boric acid (pH 8.5) for 20 minutes at room temperature. After washing in 0.3% PBST, sections were then blocked with 10% goat serum for 1 hour followed by incubation in primary antibodies for mouse anti‐BrdU (Abcam, Cambridge, UK), neural/glial antigen 2 (NG2), and/or adenomatous polyposis coli (APC) for 1 hour at room temperature and then overnight at 4°C. Brain sections were washed three times in 0.3% PBST the next day and then incubated with species‐specific secondary antibodies conjugated with DyLight 488 or DyLight 594 (Jackson ImmunoResearch Laboratories, West Grove, PA).

### 2.5 Quantification of immunofluorescent images

All immunofluorescent images were captured by an investigator blinded to experimental group assignment. To calculate the ratio of SMI32 immunostained cells to MBP immunostained cells, microscopic fields were acquired from corpus callosum (CC), cortex (CTX), and striatum (STR) in the ipsilateral hemisphere. To assess axonal trafficking, immunostaining for amyloid precursor protein (APP) was also examined in CC, CTX, and STR. The regions of interest in the sham brain and contralateral hemisphere corresponded to sites on the ipsilateral side. Parameters for acquiring the images were identical for each brain slice at the same region of interest for both hemispheres. The fluorescence intensity of MBP, SMI32, and APP was measured using ImageJ analysis software by an observer blinded to experimental group assignment. The fluorescence intensity in the ipsilateral hemisphere was further normalized to the contralateral hemisphere. The number and area of MBP^+^ or SMI32^+^ nerve fiber bundles were calculated using ImageJ analysis software by a blinded observer. Cells that were BrdU^+^/NG2^+^, BrdU^+^/APC^+^ or Iba1^+^ were counted unbiasedly by an investigator blinded to experimental group assignment. Data are expressed as average number of cells in per square millimeter.

### 2.6 Electrophysiology

Compound action potential was recorded in corpus callosum according to previously described.^39^ Rats were anesthetized with 3% isoflurane, and brains were rapidly removed. Transverse slices (350 µm) were cut on a vibrating microtome (VT1000S, Leica Biosystems, Buffalo Grove, IL) and placed in artificial cerebrospinal fluid (aCSF) saturated with 95% O_2_/5% CO_2_. Prior to use, slices were allowed to equilibrate for a half hour at 34°C followed by 1‐hour incubation at 26°C. The aCSF contained 124 mmol/L NaCl, 2.5 mmol/L KCl, 2 mmol/L CaCl_2_, 1 mmol/L NaH_2_PO_4_, 24 mmol/L NaHCO_3_, 1.3 mmol/L MgSO_4_, and 10 mmol/L D‐glucose. Recordings were performed at 26°C. To record the compound action potential (CAP) at the external capsule (EC), a concentric bipolar stimulating electrode (250 µm inner pole diameter, FHC, Bowdoin, ME) was lowered into the cingulum of CC, and a recording electrode (aCSF‐filled glass micropipette; resistance 6‐8 MΩ) was placed in EC 1.0 mm from the stimulating electrode. The initial depth of the electrodes was 100 µm below the surface of the slice. Fine adjustments were made in the depths of both stimulating and recording electrodes to optimize the signal amplitude. Evoked clausal CAP was amplified (bandpass = DC to 10 kHz), digitized at 25 kHz, and stored on a disk for offline analysis. To record the CAP at CC, the stimulating electrode was localized at the midline of CC and the recording electrode was placed in CC 1.5 mm from the stimulating electrode in both hemispheres. The stimulus intensity ranged from 0.1 to 2.0 mA, and the amplitudes of N1 and N2 were calculated using Clampfit software by an investigator blinded to experimental conditions.

### 2.7 Statistical analysis

All data are reported as mean ± SD. Differences in means across multiple groups were analyzed by one‐way ANOVA. Differences in means across multiple groups with several measurements over time were analyzed using two‐way ANOVA with repeat measurements. The post hoc Bonferroni test was used for multiple comparisons across means. Pearson's product linear regression analysis was used to correlate the white matter histological or functional parameters with performance in the foot fault test (foot fault percentage) and Morris water maze (spatial memory, time percentage spent in the target quadrant) test. *P* ≤ 0.05 was considered statistically significant.

## 3 RESULTS

### 3.1 HPC reduces brain atrophy and long‐term neurological deficits induced by H/I

**Figure 1:**
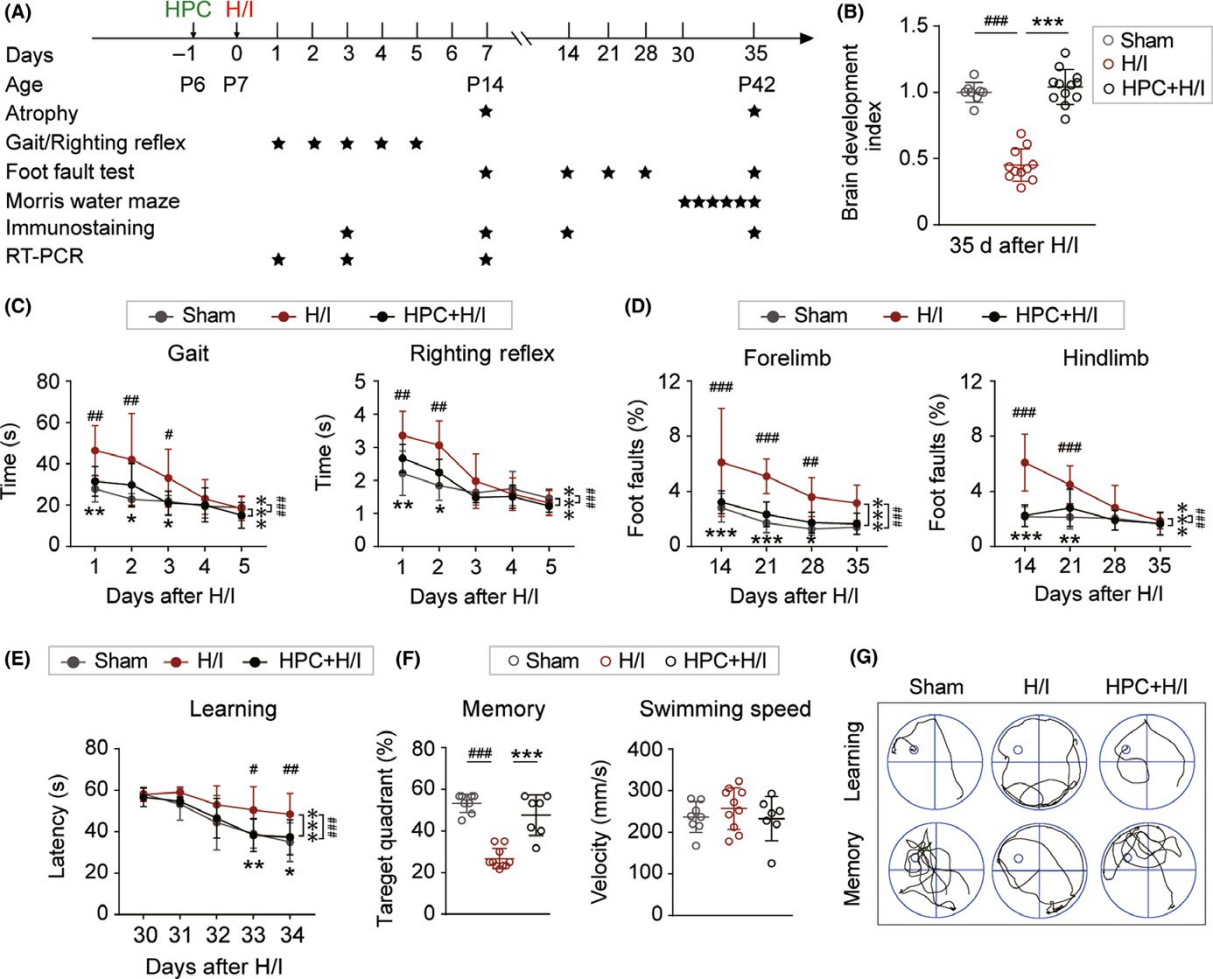
Hypoxic preconditioning (HPC) alleviates H/I‐induced brain atrophy and long‐term neurological deficits. A, Illustration of experimental timeline. B, Quantification of brain development index at 35 d after hypoxia–ischemia (H/I). Data were expressed as fold to sham. n = 9‐18 for each group. ^***^ *P* < 0.001, vs H/I group, ^###^ *P* < 0.001 vs Sham group using one‐way ANOVA followed by Bonferroni's post hoc test. C, Short‐term neurological deficits as assessed by gait and righting reflex from 1 to 5 d after H/I injury. ^#^ *P* < 0.05, ^##^ *P* < 0.01, ^###^ *P* < 0.001 vs Sham group; ^*^ *P* < 0.05, ^**^ *P* < 0.01, ^***^ *P* < 0.001 vs H/I group using one‐way or two‐way ANOVA followed by Bonferroni's post hoc test, n = 9‐18 rats per group. D, Foot fault percentage of forelimb and hindlimb. n = 7‐10 rats per group. ^##^ *P* < 0.01, ^###^ *P* < 0.001 vs Sham group, ^*^ *P* < 0.05, ^**^ *P* < 0.01, ^***^ *P* < 0.001, vs H/I group using one‐way or two‐way ANOVA followed by Bonferroni's post hoc test. E, Learning ability was reflected by latency to the target platform 30‐34 d after H/I injury in Morris water maze. F, Spatial memory was recorded at 35 d after H/I injury by measuring the time spent in the target quadrant (% of total 60 s), locomotor activities as reflected by swimming speed after H/I. n = 7‐10 rats per group. ^##^ *P* < 0.01, ^###^ *P* < 0.001 vs Sham group, ^*^ *P* < 0.05, ^**^ *P* < 0.01, ^***^ *P* < 0.001, vs H/I group using one‐way followed by Bonferroni's post hoc test. G, Representative swimming traces of rats attempting to find a hidden platform (top traces, “learning”), and searching for the platform after being removal (bottom traces, “memory”). All data are presented as mean ± SD

To assess the neuroprotective effect of HPC after H/I injury, P6 rat pups were subjected to 3 hours of hypoxia (7.8% O_2_, 92.2% N_2_) or normoxia (21% O_2_) 24 hours before surgery. P7 rat pups underwent H/I injury or sham surgery (Figure 1A). Cresyl violet staining was used to visualize tissue loss and cell morphology 7 and 35 days after injury. No significant differences were detected between hypoxia and normoxia in the sham group (data not shown). A large number of dying neurons, characterized by shrunken profiles and pyknotic nuclei, were observed in the injured CTX and STR in H/I injured neonates. As expected, HPC significantly attenuated H/I‐induced brain atrophy, reflected by less tissue loss and normalized brain development index (relative to sham brain) (Figures 1B and S1A,B).

Short‐term sensorimotor dysfunctions, as assessed with gait and righting reflex 1‐5 days after H/I injury or sham operation, were observed in H/I injured neonates (Figure 1C). HPC significantly attenuated H/I‐induced acute sensorimotor dysfunction, as shown by less time taken to move out of the target circle and to turn over to the prone position compared to H/I injured neonates. No significant differences were detected in neurological function between the two sham groups (data not shown). Altogether, the morphological and neurological data reveal that HPC significantly reduces short‐term gray matter injury and ameliorates H/I‐induced acute sensorimotor dysfunction.

Long‐term neurological function was assessed by the foot fault test and Morris water maze up to 35 days after H/I. No significant differences were detected on total steps between the three groups (Figure S1C). However, impairment of motor function was observed in neonates after H/I injury, manifested by significantly increased forelimb and hindlimb foot fault percentage compared to sham‐operated animals (Figure 1D). HPC reduced the H/I‐induced foot fault deficit up to 35 days postinjury, indicating that HPC attenuated long‐term sensorimotor dysfunctions following H/I injury. Cognitive deficits are the most commonly occurring disabilities following H/I injury in newborns.^40^ To determine whether HPC could dampen long‐term cognitive deficits induced by H/I injury, the Morris water maze test was performed between 30 and 35 days after surgery. There was a gradual improvement in cognitive performance over time for all groups. The latency to find the platform was longer in neonates that suffered from H/I injury than the neonates in the other two groups; no significant differences were detected between sham and HPC + H/I (Figure 1E). Compared with neonates in the H/I group, HPC‐treated rats swam across the target area more frequently, and spent more time in the target quadrant, when the platform was removed during the probe trial (Figures 1F and S1D). There was no significant difference in swimming velocity among the three groups, which suggests that decreased latency to the target platform was not due to H/I‐induced muscle atrophy (Figure 1F,G).

### 3.2 Delayed brain development is revitalized by HPC following H/I injury

**Figure 2:**
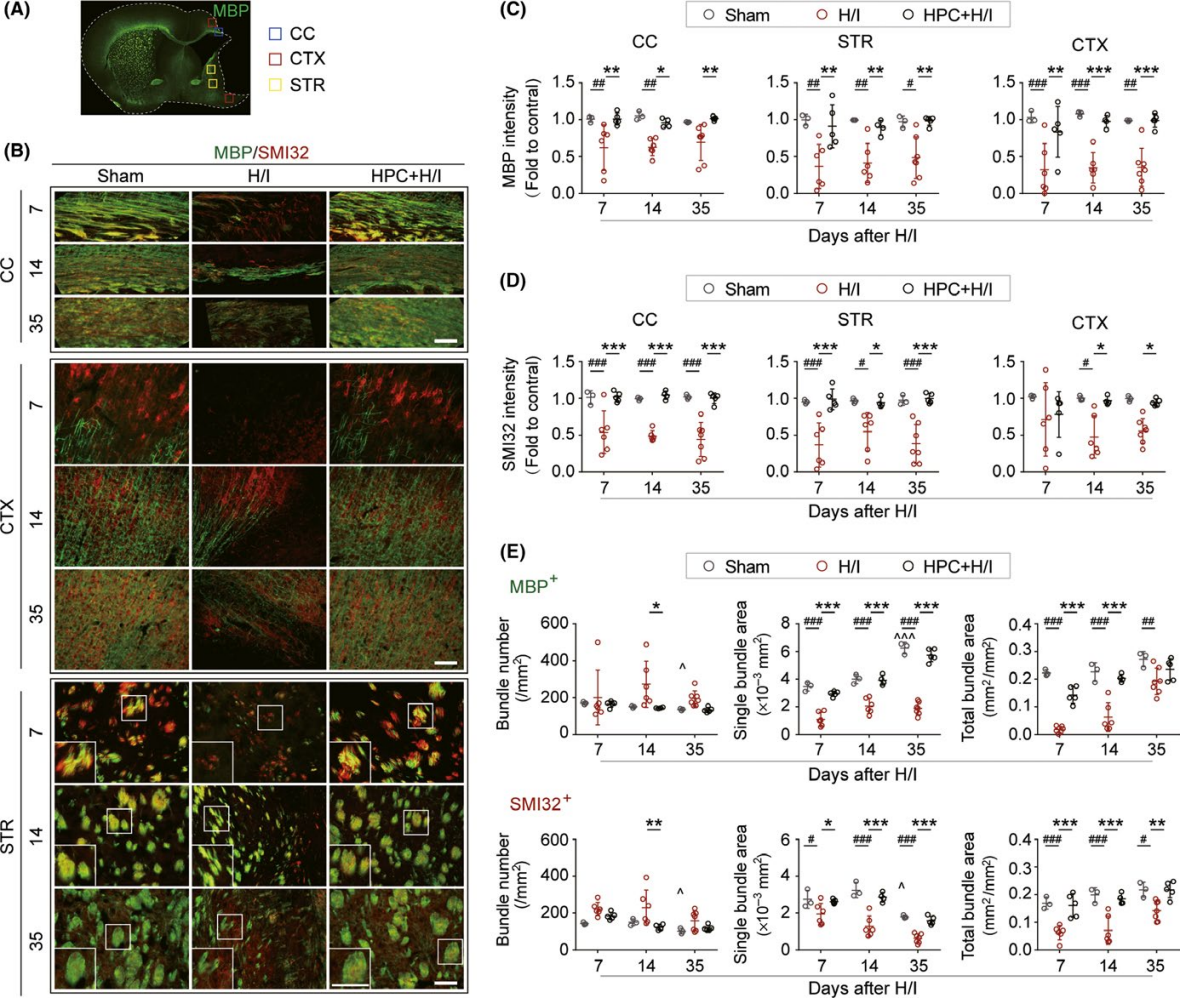
Hypoxic preconditioning (HPC) revitalizes brain development delayed by hypoxia–ischemia (H/I). A, Representative low‐power image of MBP (green) staining in the corpus callosum (CC), cortex (CTX) and striatum (STR) (B) Immunofluorescence double‐labeled staining of MBP and SMI32 in CC, CTX, and STR of the ipsilateral hemisphere at indicated time points after H/I injury. The white boxes showed enlarged regions in striatum. Scale bar = 100 μm. Quantification of the relative ratio of MBP (C) and SMI32 (D) intensity of ipsilateral to contralateral hemisphere in the corresponding areas in CC, CTX, and STR at indicated time points after H/I injury. n = 3‐7 rats per group at each time point. E, Quantification of MBP^+^ (up panel) or SMI32^+^ (bottom panel) total bundle number (first column), single bundle area (second column), and total bundle area (third column) in striatum. ^#^ *P* < 0.05, ^##^ *P* < 0.01, ^###^ *P* < 0.001 vs Sham group, ^*^ *P* < 0.05, ^**^ *P* < 0.01, ^***^ *P* < 0.001, vs H/I group, ^^^ *P* < 0.05, ^^^^^ *P* < 0.001 vs sham at 7 d, analyzed using one‐way ANOVA followed by Bonferroni's post hoc test. All data are presented as mean ± SD

Hypoxia/ischemia‐induced perinatal brain injury results in progressive damage to white matter in the developing brain.^13^, ^41^ To investigate whether HPC could attenuate H/I‐induced impairment on white matter integrity, SMI32 (red), a marker for nonphosphorylated axons, and MBP (green), a marker of mature myelin, were stained to visualize the development of axons and myelination, respectively, after H/I injury (Figure 2A). In sham‐operated neonatal rats, MBP expression was dramatically increased and widely detected in CC, CTX, and STR from 7 to 35 days after surgery. The increased co‐localization of MBP and SMI32 in the sham group showed the progression of myelin formation in the developing brain. However, the fluorescent intensity of both MBP and SMI32 significantly decreased up to 35 days after H/I, indicating the dysplasia of white matter after H/I injury. HPC restored the development of white matter almost back to normal levels, as shown by equivalent fluorescent intensity of MBP and SMI32 in all regions of interest between sham and HPC + H/I animals (Figure 2B‐D).

To further examine the effect of HPC on myelin integrity after H/I injury, MBP^+^ or SMI32^+^ nerve fiber bundles were, respectively, assessed for bundle number and area in striatum after H/I injury. In sham‐operated rats, myelin formation and maturation was characterized by a decreased number of MBP^+^ bundles, increased MBP^+^ single bundle area, and increased total bundle area from 7 to 35 days (Figure 2E marked by **^)**. Although H/I injury did not induce a significant change in the number of MBP^+^ bundles, the MBP^+^ single bundle area and total bundle area remarkably decreased after H/I injury. HPC restored the stability of MBP^+^ bundles (Figure 2B,E). Similar changes were detected in SMI32^+^ bundles up to 35 days after surgery, suggesting that development of white matter was severely disrupted, but was effectively revived by HPC (Figure 2B,E). NF200, a marker of phosphorylated neurofilament, was also dramatically decreased in the investigated region of interest, and the data showed that HPC preserved the levels of NF200 immunofluorescence at 35 days after H/I (Figure S2).

The effect of HPC on axonal trafficking was determined by immunofluorescence staining of APP, which was physically transferred from the neuronal body to the axon terminals. APP^+^ immunofluorescent signal was scarcely visible in sham brain. However, APP largely accumulated in peri‐lesion areas of the CC, CTX, and STR in the ipsilateral hemisphere after H/I injury and was significantly suppressed by HPC (Figure S3A,B).

### 3.3 Long‐term neurological outcomes are correlated with white matter integrity

**Figure 3:**
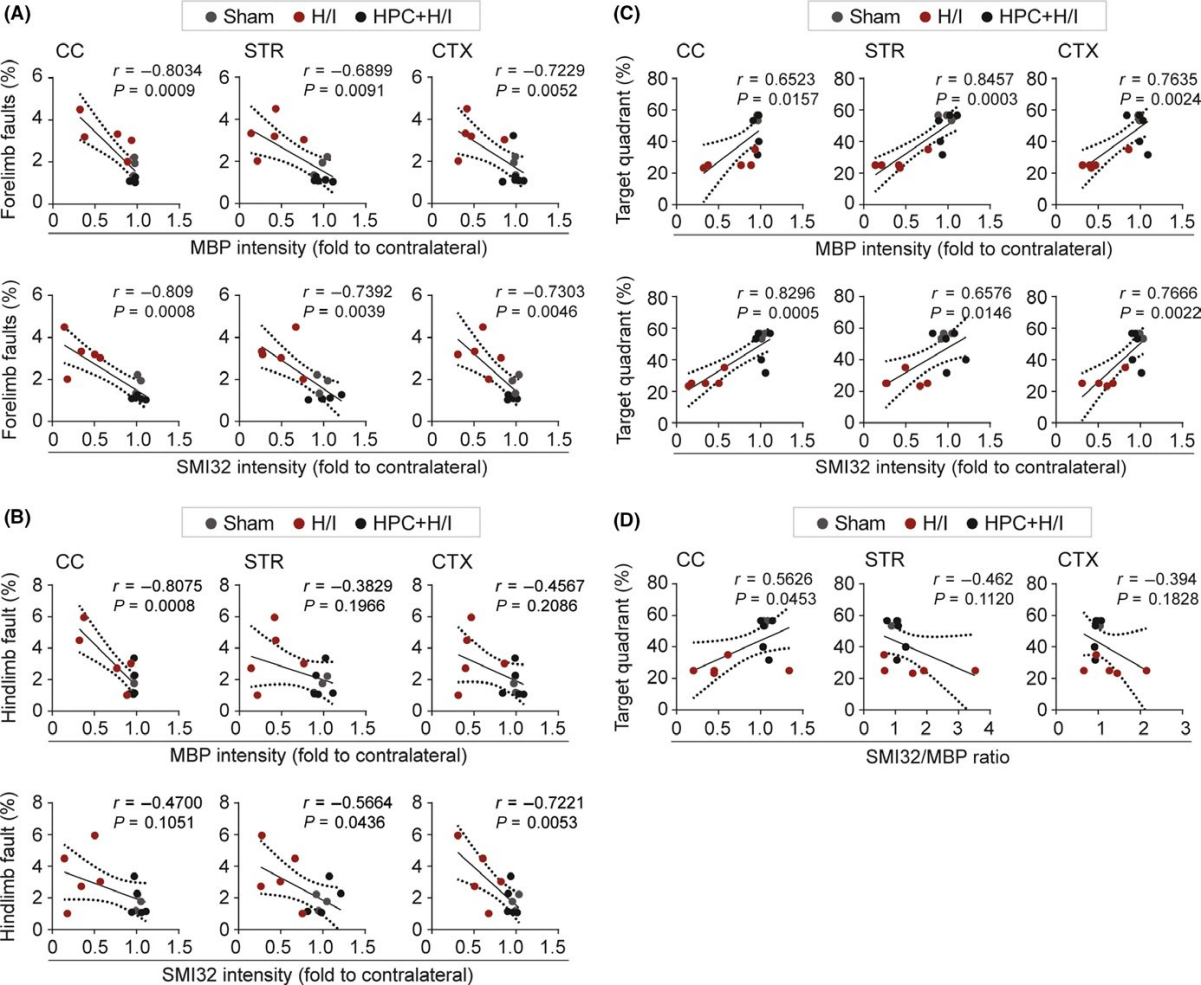
White matter integrity is associated with recovery of long‐term neurological outcomes after hypoxia–ischemia (H/I). Pearson product linear regression analysis was performed to correlate white matter histological parameters with the performance in the foot fault test (A and B) and Morris water maze test (C and D) after H/I injury. A, Correlation of MBP intensity or SMI32 intensity in CC (first column), STR (second column), or CTX (third column) regions marked in Figure 2A 35 d after H/I injury with forelimb fault percentage (A) or hindlimb fault percentage (B) in the foot fault test, as well as spatial memory (the percentage of time spent in the target quadrant) in the Morris water maze (C). D, Correlation of SMI32/MBP ratio in the indicated region (CC, STR, and CTX) with spatial memory (the percentage of time spent in the target quadrant) in Morris water maze. n = 3‐5 rats per group

Normal development of the brain may contribute to long‐term neurological functional recovery. However, normal brain development is retarded after neonatal H/I brain injury. To ascertain whether improvement in the cerebral microstructure (MBP^+^ intensity, SMI32^+^ intensity, and SMI32/MBP ratio) correlated with attenuation of long‐term sensorimotor and cognitive deficits, Pearson's product regression analysis was performed. Strong negative correlations were found between white matter integrity in CC, STR, and CTX and foot fault 35 days after H/I injury, as reflected by improved MBP^+^ intensity and decreased foot fault (forelimb and hindlimb) percentage (Figure 3A,B). Improvement in white matter integrity also correlated significantly with enhanced cognitive function (time percentage spent in target quadrant) in all three brain regions examined 35 days after surgery (Figure 3C). Furthermore, SMI32^+^ intensity in CC, STR, and CTX was negatively correlated with foot fault percentage, and positively correlated with cognitive function (Figure 3A‐C). No significant correlation was observed between the SMI32/MBP ratio and foot fault percentage (Figure S4). In regard to the relation between the SMI32/MBP ratio and cognitive function, marginal correlation was found only in CC, but not in STR and CTX (Figure 3D).

Pearson's correlation analysis was further performed to assess the association between the severity of axonal damage and long‐term neurological outcomes in the foot fault test and Morris water maze. APP intensity in CC and STR was only correlated with the forelimb fault percentage 35 days after H/I injury (Figure S3C,D). Moreover, a negative correlation was detected between axon injury and cognitive function in the Morris water maze, as reflected by increased APP intensity in CC, STR, and CTX, and worse performance in the probe test 35 days after H/I injury (Figure S3E).

### 3.4 HPC reverses H/I‐induced abnormal proliferation and maturation of OPCs

**Figure 4:**
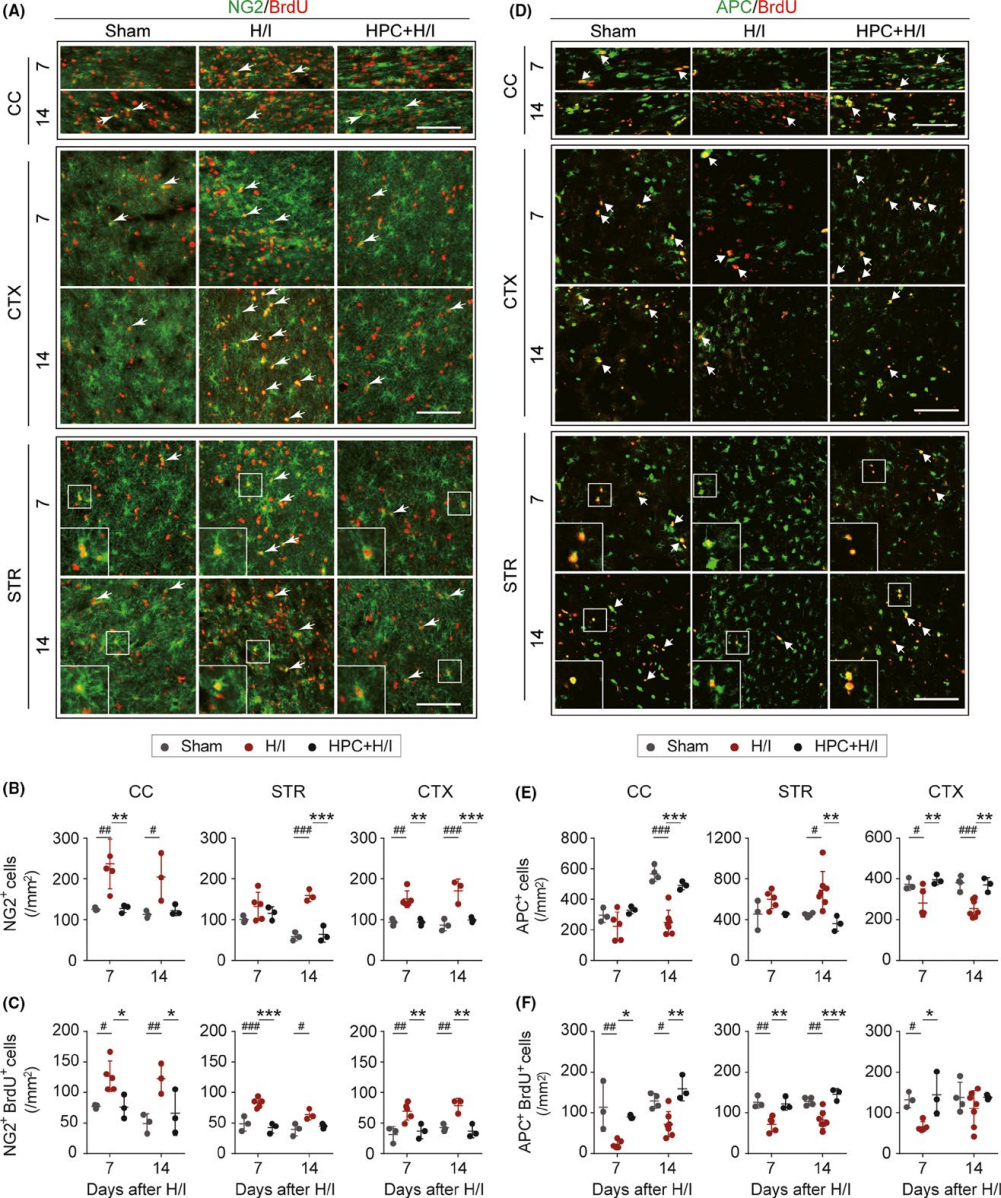
Hypoxic preconditioning (HPC) sustains oligodendrocyte progenitor cell maturation after neonatal hypoxia–ischemia (H/I) brain injury. A‐C, Immunofluorescent staining of NG2 (green) and BrdU (red) in corpus callosum (CC), cortex (CTX) and striatum (STR) of ipsilateral hemisphere 7 and 14 d after H/I injury. The white boxes illustrate the co‐localization of NG2 and BrdU in enlarged areas of STR. Arrowhead: NG2^+^/BrdU^+^ cells. Scale bar = 100 μm. Quantification of NG2^+^ cells (B) and NG2^+^/BrdU^+^ cells (C) in per mm^2^ in CC, STR, and CTX 7 and 14 d after H/I injury. D, Representative images of APC (green) and BrdU (red) in CC, CTX, and STR of ipsilateral hemisphere 7 and 14 d after neonatal H/I brain injury. Arrowhead: APC^+^/BrdU^+^ cells. The white boxes illustrate the area of high‐power images of APC and BrdU double labeling at the left corner in striatum. Scale bar = 100 μm. Quantification of APC^+^ cells (E) and APC^+^/BrdU^+^ cells (F) in per mm^2^ in CC, STR, and CTX 7 and 14 d after H/I injury. n = 3‐7 rats per group. ^#^ *P* < 0.05, ^##^ *P* < 0.01, ^###^ *P* < 0.001 vs Sham group, ^*^ *P* < 0.05, ^**^ *P* < 0.01, ^***^ *P* < 0.001, vs H/I group using one‐way ANOVA followed by Bonferroni's post hoc test. All data are presented as mean ± SD

Oligodendrocyte progenitor cell proliferation and maturation are crucial during development for the formation of myelin and can counteract the secondary loss of axons and neurons caused by injury‐induced retrograde degeneration. To determine the effect of HPC on the proliferation and maturation of OPCs after H/I injury, double staining of NG2 and BrdU, or APC and BrdU was performed and quantified in CC, STR, and CTX at 7 and 14 days after H/I injury (Figure 4A,D). The number of NG2^+^ and NG2^+^/BrdU^+^ cells (white arrows indicated) was significantly increased in H/I injured rats in the three brain regions examined compared to sham. HPC prevented the abnormal accumulation of OPCs induced by H/I injury (Figure 4B,C). OPCs give rise to mature oligodendrocytes for the development of myelin. Thus, we assessed the genesis of mature oligodendrocytes using APC/BrdU immunostaining (Figure 4D). Newly generated oligodendrocytes (APC^+^/BrdU^+^ cells, white arrows indicated) were significantly decreased in all regions of interest after surgery, suggesting that maturation of OPCs was potentially impeded by H/I injury. In contrast to the loss of mature oligodendrocytes observed in CC and CTX, H/I injury resulted in a dramatic increase in APC^+^ cells in the STR 14 days after surgery. HPC preserved the proliferation and maturation activities of OPCs, as well as the survival of mature oligodendrocytes (Figure 4E,F). Collectively, these findings suggest that H/I injury stimulated proliferation of OPCs, but impeded their differentiation and maturation in the developing brain. This phenomenon was effectively reversed by HPC.

### 3.5 HPC confers long‐term effects on myelin formation and maturation following H/I injury

**Figure 5:**
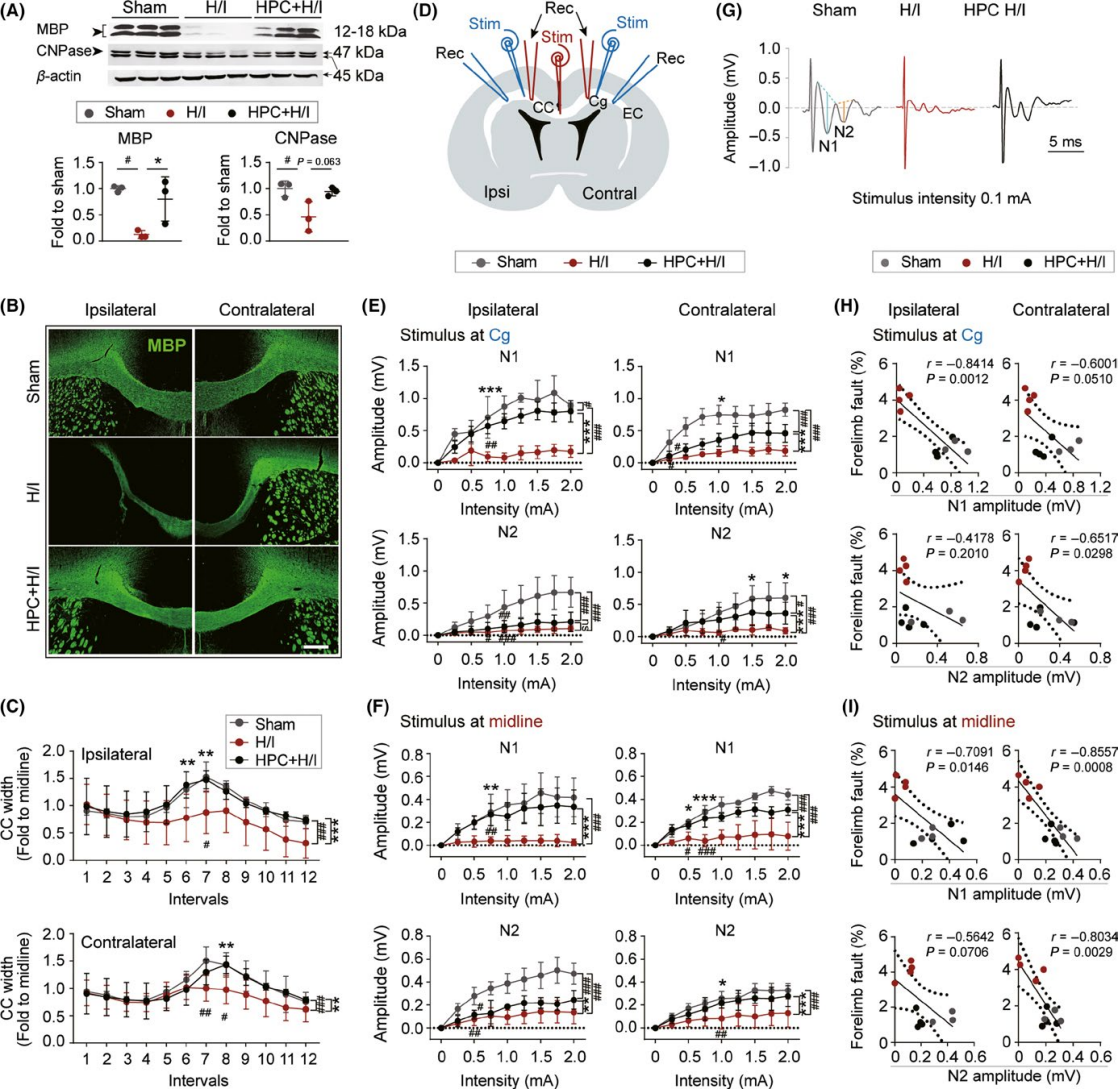
Hypoxic preconditioning (HPC) preserves myelin formation and maturation after hypoxia–ischemia (H/I) injury. A, Representative western blot images of MBP and CNPase in lesion ipsilateral hemisphere 35 d after H/I injury. Semi‐quantification of bands relative to sham rats. n = 3 rats per group. ^#^ *P* < 0.05, vs Sham, ^*^ *P* < 0.05 vs H/I group using one‐way ANOVA followed by Bonferroni's post hoc test. B, Representative images of MBP (green) staining shows the relative corpus callosum width in ipsilateral and contralateral hemisphere at 35 d after H/I injury. Scale bar = 200 μm. C, Quantification of corpus callosum (CC) width (fold to the thickness of midline) in both ipsilateral and contralateral hemisphere 35 d after H/I injury. Intervals = 80 μm. n = 3‐6 per group. ^#^ *P* < 0.05, ^##^ *P* < 0.01, ^###^ *P* < 0.001 vs Sham group, ^**^ *P* < 0.01, ^***^ *P* < 0.001 vs H/I group using one‐way and two‐way ANOVA followed by Bonferroni's post hoc test. D, Illustration of the position of stimulating (at Cg, cingulate gyrus, blue; at midline, red) and recording electrodes in the CC 35 d after H/I or Sham surgery. E and F, Quantification of the N1 (top panel) and N2 (bottom panel) amplitudes in the ipsilateral (left panel) and contralateral (right panel) hemispheres. E, Stimulating electrode was placed at Cg, and the response was recorded in the same hemisphere 1 mm lateral to the stimulation point. F, Stimulating electrode was localized at midline, and the response was recorded along both sides of the CC. The distance between stimulating and recording electrodes was 1.5 mm. n = 3‐4 rats per group. ^#^ *P* < 0.05, ^##^ *P* < 0.01, ^###^ *P* < 0.001 vs Sham group, ^*^ *P* < 0.05, ^**^ *P* < 0.01, ^***^ *P* < 0.001 vs H/I group using two‐way ANOVA followed by Bonferroni's post hoc test. G, Representative traces of evoked CAP in CC (stimulus 0.1 mA, recording at 1.0 mm lateral to the stimulating electrode). H and I, The amplitude of N1 and N2 recorded at ipsilateral (left panel) and contralateral (right panel) CC in response to 0.1 mA stimulus was correlated with forelimb fault percentage (% of total steps) in foot fault test 35 d after H/I injury by Pearson's product linear regression analysis. H, Stimulus at Cg. I, Stimulus at midline. n = 3‐4 rats per group. All data are presented as mean ± SD

Hypoxic preconditioning sustained normal brain development by restoring the proliferation and maturation of OPCs at early time points after H/I injury. To determine whether HPC has long‐term effects on the integrity of white matter, the protein expression of MBP and CNPase in mature oligodendrocytes and the width of CC were analyzed 35 days after H/I injury. Levels of MBP and CNPase were remarkably suppressed in the ipsilateral hemisphere after injury. Exposure to hypoxic conditions prior to H/I injury prevented the decrease in MBP protein expression observed at 35 days with H/I injury alone (Figure 5A). Examination of the CC, where myelinated axons are enriched, showed that H/I injury significantly reduced relative callosal width (compared with the width of midline) in both hemispheres 35 days after H/I injury, and that this reduction was prevented by HPC (Figure 5B,C).

Myelin sheath is a key contributor to efficient and rapid signal conduction along myelinated axons within the CNS. To assess whether newly generated myelin is functional, evoked CAP was recorded in the CC in both hemispheres 35 days after H/I injury. Generally, a biphasic waveform was recorded, comprising an initial segment (N1, representing fast‐conducting myelinated axons), followed by a second segment (N2, representing the slower‐conducting unmyelinated axons). Amplitudes of N1 and N2 were used to reflect the signal conductivity of myelinated and unmyelinated axons, respectively (Figure 5G). Two stimulus points (Stim) were chosen for CAP recordings (Figure 5D). When the stimulus was positioned at Cg of EC, a significant decrease in N1 and N2 segments was found in both the ipsilateral and contralateral CC in the H/I injured group, indicating reduced conductivity of both myelinated and unmyelinated axons in whole brain. HPC‐treated rats showed a robust increase in N1 amplitude, but not N2 amplitude, on the ipsilateral side (Figure 5E). Both N1 and N2 amplitudes were significantly increased by HPC in the contralateral EC. When the stimulus was positioned at the midline of CC, HPC treatment was still able to robustly restore signal conductivity in both myelinated and unmyelinated axons, as revealed by the increase in N1 and N2 segments in both hemispheres (Figure 5F). In addition, Pearson's correlation analysis showed negative correlation between the amplitude of N1 and forelimb fault when recorded on both sides of the CC. No significant correlation was detected between forelimb fault and N2 in the ipsilateral hemisphere (Figure 5H). The amplitude of N1 and N2 recorded from the CC with the stimulus at the midline was highly negatively correlated with performance on the forelimb fault test 35 days after H/I injury (Figure 5I). Thus, HPC treatment sustains normal morphological and functional development of both white matter after H/I injury, as reflected by restored conductivity of unmyelinated and myelinated axons, all of which contributed to long‐term neurological functional recovery.

### 3.6 HPC reduces microglia/macrophages activation and cytokine production after H/I injury

**Figure 6:**
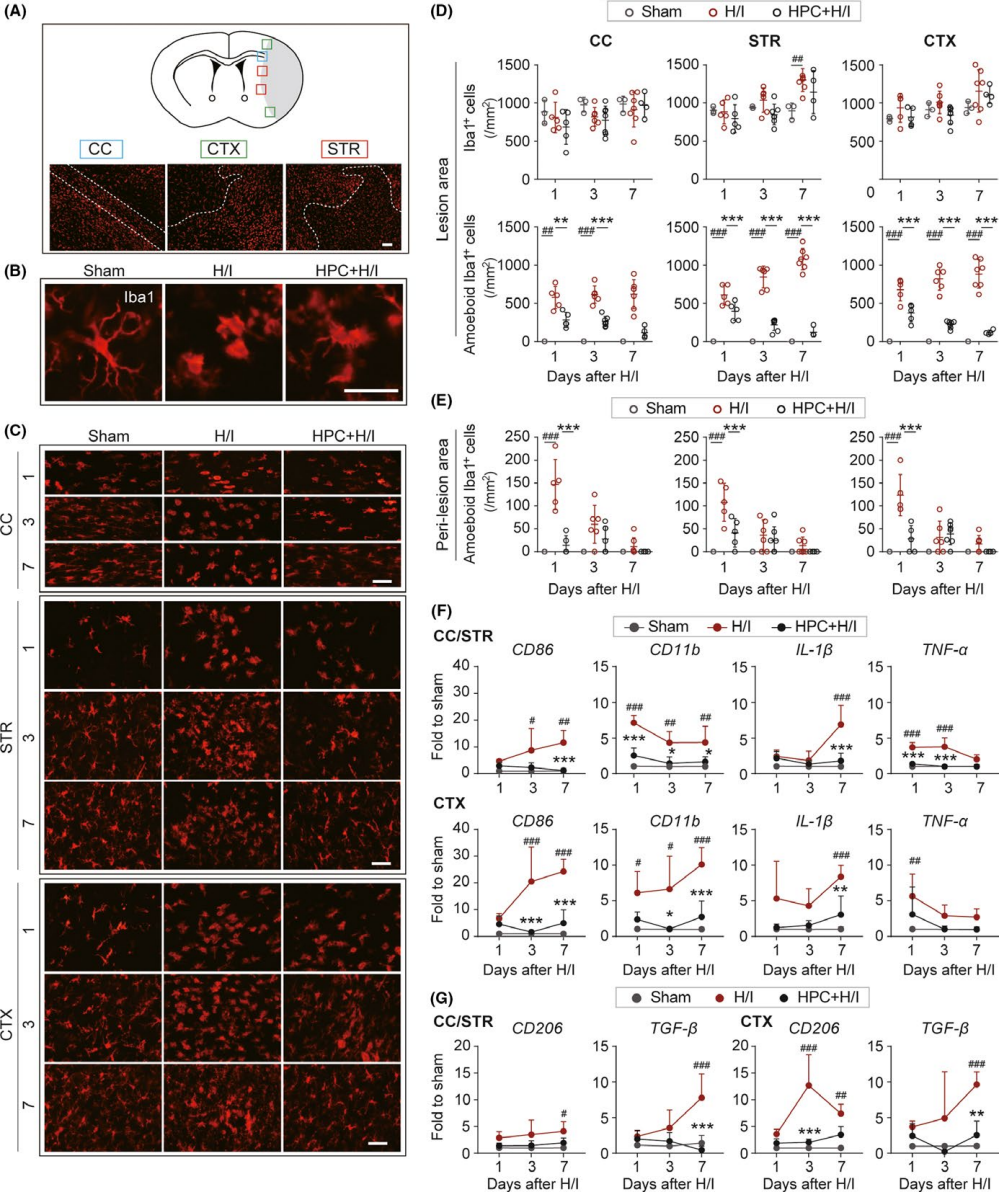
Hypoxic preconditioning (HPC) ameliorates H/I‐induced microglia activation and inflammatory cytokines expression. A, The schematic graph of coronal brain section (top panel). The color boxes illustrate the corpus callosum (CC), cortex (CTX) and striatum (STR) areas in ipsilateral hemisphere after hypoxia–ischemia (H/I) injury where images were taken. Representative images of Iba1 (red) immunofluorescent staining in CC, CTX, and STR (bottom panel). The dashed lines depict the border of lesion area and peri‐lesion area. Scale bar = 100 μm. B, Representative images of Iba1 showed the morphology of resting microglia in the sham group and the activated microglia/macrophages (ameboid microglia/macrophages) in the H/I groups after injury. Scale bar = 10 μm. C, Immunofluorescent staining of Iba1 (red) in CC, STR, and CTX in the ipsilateral hemisphere 1, 3, and 7 d after H/I injury. Scale bar = 100 μm. D, Quantification of the total Iba1^+^ cells (up panel) and ameboid (activated) Iba1^+^ cells (bottom panel) in lesion areas of CC, STR, and CTX 1, 3, and 7 d after H/I. E, Quantification of the activated Iba1^+^ cells at peri‐lesion areas of CC, STR, and CTX 1, 3, and 7 d after H/I. n = 3‐5 rats per group. ^##^ *P* < 0.01, ^###^ *P* < 0.001 vs Sham group, ^**^ *P* < 0.01, ^***^ *P* < 0.001 vs H/I group using one‐way ANOVA followed by Bonferroni's post hoc test. F and G, RT‐PCR analysis of microglia/macrophage pro‐inflammatory cytokine (F) and inflammation‐resolving cytokines (G) of damaged CC/STR and CTX brain tissue 1, 3, and 7 d after H/I injury. n = 3‐4 rats per group. ^#^ *P* < 0.05, ^##^ *P* < 0.01, ^###^ *P* < 0.001 vs H/I group using one‐way ANOVA followed by Bonferroni's post hoc test. All data are presented as mean ± SD

Microglia/macrophages are intricately involved in the initiation and propagation of the inflammatory response to CNS injury. To investigate whether the beneficial effects of HPC on white matter integrity involved modulation of the inflammatory response, microglia/macrophage activation was measured using an antibody for Iba1, a microglia/macrophage marker. Microglia/macrophage was activated from the resting state to an activated state, showing an ameboid morphology (Figure 6A‐C) in response to H/I injury. The total number of Iba1^+^ cells gradually increased in the injured area of the H/I group compared with the sham and the HPC + H/I groups. However, significant difference was only found in the STR between the sham and H/I injured animals 7 days after H/I injury. When comparing the number of ameboid activated Iba1^+^ cells within lesion areas, there was a significant increase in ameboid activated microglia/macrophage after H/I injury in CC, STR, and CTX, which was abated up to 7 days in neonates that underwent HPC (Figure 6D). Similar results were obtained in the peri‐lesion area of the CC, STR, and CTX 1 day after H/I injury (Figure 6E).

Expression of pro‐inflammatory cytokines, including TNF‐α, CD11b, CD86, and IL‐1β, was increased 1, 3, and 7 days in the injured CC/STR and CTX after H/I injury. However, exposure to hypoxic conditions prior to H/I injury prevented the increase in these pro‐inflammatory cytokines (Figure 6F). No significant difference was found between HPC‐treated animals and sham surgery rats. Coincidentally, HPC also prevented the upregulation of the anti‐inflammatory cytokines, CD206, and TGF‐β after H/I injury (Figure 6G). In summary, HPC‐induced alleviation of microglia/macrophage activation and suppression of inflammatory cytokine expression may partially contribute to normal development of the brain after H/I injury.

## 4 DISCUSSIONS

The present study is the first to demonstrate that HPC promotes long‐term neurological functional outcomes after H/I injury and counteracts H/I‐induced developmental retardation of the immature brain, which is associated with restored differentiation and maturation of oligodendrocyte progenitors into myelin‐generating oligodendrocytes and inhibited inflammatory responses in microglia and macrophages. The beneficial effects of HPC on hypoxic‐ischemic injured neonatal rats have been reported for many years. Although the underlying mechanisms are still not fully understood, several theories have been put forward. One such theory is that sublethal hypoxia upregulates several pro‐survival proteins, such as HIF1, Hsp, BDNF, and Bcl‐2, which then promote neuronal survival after cerebral ischemia or hypoxia.^42^, ^43^ However, neuronal survival in itself is not sufficient to protect the immature brain against H/I injury, as white matter injury is considered to be the leading cause of lifelong neurological deficits in survivors after H/I injury.^13^

White matter injury is characterized by perturbations in myelination that are initiated by acute death of premyelinating oligodendrocytes and chronic failure of normal maturation of OPCs.^11^, ^44^, ^45^ Previous examination into the effect of HPC on white matter injury has been very limited. Suryana et al first reported attenuation in the loss of myelin by HPC 5 days post‐H/I injury, but they did not find any changes in the number of early and late OPCs in CC.^35^ In our study, HPC exerted long‐term effects on postnatal development of the brain parenchyma and enhanced neurological functional recovery after H/I injury. Deterioration of axons after H/I injury resulted in long‐term sensorimotor dysfunctions, as shown by the correlation between SMI32^+^ and functional performances. However, diminution of long‐term neurological deficits was not only attributed to decreases in axon damage, but also by critical effects of HPC on myelination, which was supported by significant correlation between MBP^+^ intensity and long‐term neurological outcomes. Thus, axonal myelination produced by oligodendrocytes was critical to functional recovery in the developing brain following H/I injury.

Restoration of white matter structure contributed to the functional integrity of white matter and to the recovery of neurological function.^7^, ^46^ OPCs were essential for the replacement of degenerating premyelinated oligodendrocytes and to the development of new myelin in the immature brain after H/I injury.^13^ As the major component of white matter, oligodendrocyte lineage cells possess different sensitivities to H/I insult. The late OPCs are more vulnerable to H/I insult relative to early OPCs or mature oligodendrocytes in the developing brain. The loss of late OPCs caused by activation of caspase‐3 or accelerated maturation to reactive oligodendrocytes results in myelination dysfunction and prolonged atrophy of white matter.^9^, ^13^ Besides being prone to degeneration, newly generated OPCs can suffer from maturation arrest, which may predispose the developing brain to chronic white matter injury after H/I injury.^44^ Previous research suggested that HPC had no significant impact on either the number of early or late OPCs in white matter after H/I injury.^35^ Herein, we demonstrate that H/I injury stimulated the proliferation and accumulation of NG2^+^ early OPCs. However, the maturation of OPCs was dramatically delayed in affected regions of the CC and CTX, as reflected by decreased APC^+^ and APC^+^/BrdU^+^ cells in H/I injured neonatal rats. The maturation arrest that occurred in OPCs may be related to inhibitory factors derived from reactive astrocytes or microglia/macrophages.^11^, ^23^ The decline in the number of APC^+^ cells may also be attributed to death of mature oligodendrocytes induced by H/I injury. The aforementioned pathological changes were dramatically reversed by HPC. In STR, H/I injury reduced the number of newly generated mature oligodendrocytes (APC^+^/BrdU^+^ cells), but the total number of APC^+^ cells was significantly increased 14 days after H/I injury. The increase in the number of APC^+^/BrdU^‐^ cells may derive from the survival of OPCs rather than newly generated ones (NG2^+^/BrdU^+^), suggesting that H/I injury may potentially stimulate OPC maturation to replace injured mature oligodendrocytes. However, these reactive oligodendrocytes may not effectively execute myelination activity,^9^ as shown by the persistent deficiency of MBP intensity in STR up to 35 days after H/I injury. Thus, HPC preserved normal proliferation and maturation activities of OPCs in H/I injured rats.

White matter is comprised of myelin sheath and axons. CC is the largest white matter tract in the brain. The CC contains interhemispheric functional connectivity that is critical for higher order functions, including motor activities and memory.^47^ The link between the size of corpus callosum and motor development has been documented in preterm and term infants.^48^, ^49^ The current study demonstrated a close correlation between the expression of white matter markers (MBP or SMI32) and sensorimotor deficits, suggesting that white matter integrity is important for sensorimotor performance of the H/I‐injured neonates. This observation is consistent with previous studies in several neurological diseases, which reported correlations between CC disruption and cognitive or memory dysfunction.^50^, ^51^, ^52^ Surprisingly, SMI32/MBP ratio, an excellent parameter for white matter integrity in adult brain,^53^ was poorly correlated with the cognitive performance of neonatal rats after H/I, probably due to the simultaneous loss of both myelin and axons in the neonatal brains. Therefore, either SMI32 or MBP intensity alone might be a useful parameter for white matter integrity in developing brains after H/I.

The effect of HPC on white matter functional integrity after H/I injury was assessed by measuring the CAP of myelinated and unmyelinated axons in the CC. Our results demonstrated that the functional integrity of both myelinated and unmyelinated axons was preserved in the ipsilateral CC by HPC. However, no significant differences were detected in the amplitude of N2 between the two H/I injured groups when stimulus was initiated at Cg, suggesting that unmyelinated axons may be more sensitive to H/I damage. To our surprise, brain atrophy induced by unilateral ligation of the common carotid artery in H/I‐treated animals was not restricted to the ipsilateral hemisphere, but also affected the contralateral side. HPC‐induced trophic effects on brain development also extended to the CC of both hemispheres, as manifested by restoration of CC thickness and conductivity of both myelinated and unmyelinated axons. Therefore, we conclude that the functional integrity of white matter contributed to the long‐term recovery of motor and cognitive function.

As resident immune cells, microglia are critical to the development of the brain. Perinatal H/I brain injury induces acute inflammatory responses, which could extend for weeks and even months. Activated microglia/macrophages produce inflammatory factors such as cytokines, chemokines, and reactive oxygen species, which may potentially impede the development of gray and white matter after H/I injury.^54^, ^55^, ^56^, ^57^ Inflammation‐resolving cells, also called M2 phase microglia/macrophages, have been shown to have multiple trophic effects, on the proliferation of glia progenitor and maturation of oligodendrocytes in the adult brain.^58^, ^59^ Our study demonstrates that HPC mainly suppressed activation of microglia/macrophage and cytokines production,^30^ rather than regulated microglia/macrophages polarization in the immature brain after H/I injury. Therefore, therapeutic strategies that target the activation of microglia could be potentially effective in restoring normal brain development following H/I insult.^60^

In conclusion, HPC sustained the development of white matter and gray matter in the immature brain, contributing to long‐term neurological functional recovery after H/I brain injury. HPC restored the differentiation and maturation capacities of OPCs into myelination oligodendrocytes, and reduced microglia/macrophage activation and neuroinflammation.

## CONFLICT OF INTEREST

The authors declare no conflict of interest.
