# Supplementary material for: Hypoxic preconditioning improves long‐term functional outcomes after neonatal hypoxia–ischemic injury by restoring white matter integrity and brain development
Source: CNS Neurosci Ther. 2019 Jan 28;25(6):734–47. doi: 10.1111/cns.13102 (PMC6515700; doi:10.1111/cns.13102)
Supplement: Supplementary file 1 [file CNS-25-734-s001.docx]

**Supplementary Materials**

**Material and Methods**

1. **Tissue section preparation**

Rats were anesthetized and trans-cardiac perfused with cold saline followed by cold 4% paraformaldehyde in 0.1mol/L phosphate buffer (pH 7.4). Brains were subsequently removed and continuously fixed in 4% paraformaldehyde overnight, followed by gradient dehydration into 20% and 30% sucrose solutions, respectively. Frozen serial coronal brain sections were sliced at 25μm-thickness on a cryostat (CM1900, Leica, Bensheim, Germany) and stored in storing buffer at -20°C.

1. **Nissl staining**

Brain sections were mounted onto slides and subjected to dehydration in a graded series of ethanol. After being washed in distilled water, brain slices were submerged in cresyl violet staining solution for 20 minutes and then placed in 95% ethanol (acetic acid added to a pH of 4.1) for color separation. The sections were then immersed in xylene for 5 minutes, and mounted with neutral balsam.

1. **Measurement of brain atrophy**

Coronal brain sections through the corpus callosum and dorsal hippocampus (bregma +1.1 to -2.5 mm) were obtained at 7 days or 35 days after neonatal H/I for Nissl staining and then used for histologic assessment of tissue loss as previously described.^1^ The stained brain slices were photographed (DM5000B, Leica) and the volume of both hemisphere was measured with ImageJ analysis software by an investigator blinded to the experimental conditions. The percentage of acute tissue loss was calculated according to the following formula: [(volume of contralateral hemisphere-volume of ipsilateral hemisphere)/volume of contralateral hemisphere] x 100. Brain development index assessed at 35 days after H/I was calculated according to the following formula: volume of ipsilateral hemisphere of sham /volume of ipsilateral hemisphere of H/I.

1. **Neurological function evaluation**

***Gait test***: Animals were placed in the center of a circle with 13 cm-diameter, and the time they moved out of the circle with both forelimbs was recorded. In cases the animal did not leave the circle for 60 seconds, the test was considered to be 60 seconds. ***Righting reflex***: Rats were placed on supine position and the time needed to turn over to prone position and place all four paws in contact with the surface was recorded. The maximum time is 60 seconds. ***Foot fault* *test***: Neonates were placed on a stainless steel grid floor (20 cm × 40 cm with a mesh size of 4 cm^2^) elevated 1.5 m above the floor. Total steps and foot faults were recorded for 3 minutes. Foot fault was defined as misplaced forelimb or hindlimb and fell through the grid floor. ***Morris water maze test***: Cognitive deficits were performed using Morris water maze at 30-35 days after H/I as previously described.^2^ Briefly, a black circular pool (diameter: 160 cm, height: 60 cm) was filled with clear water to depth of 30 cm at a temperature of 21±1℃. The pool was divided into 4 quadrants, and the platform (diameter: 15 cm) was submerged in a quadrant of the pool 1 cm below the surface. In learning test, rat was placed into the water facing the maze wall from one of four start positions, and was allowed to swim for 60 seconds to find the platform. The order of start positions was randomly chosen every day, and the latency to reach the submerged platform was recorded at 30-34 days after H/I. If the animals failed to find the platform within 60 seconds, they will be led to the platform and stay on it for 15 seconds. The rats were given four trials per day for five consecutive days with an inter-trial interval of 30 min. At 35 days after H/I, the platform was removed, and each rat was placed into water from the former training positions to test the memory function. The number of times the rat crossed the area where the platform used to be and the total time the rat stayed in the platform quadrant were recorded.

1. **Calculation of CC width**

The width of corpus callosum was assessed in MBP-stained sections by dividing the corpus callosum into 12 equal intervals (80 µm/interval) in Photoshop. The width of the CC was measured every 80 µm from the midline for both contralateral and ipsilateral hemispheres by a blinded observer at 35 days after H/I. Data are expressed as relative thickness to the width of midline.

1. **Western blot**

Tissue from ipsilateral hemisphere was harvested at 35 days after H/I. Western blot analysis was performed according to the standard procedure of the SDS-PAGE method as previously described.^3^ After blocking, PVDF membranes were incubated in primary antibody at room temperature for an hour and then overnight at 4°C. The next day, membranes were incubated with HRP-conjugated secondary antibody for an hour at room temperature, and ECL was used to visualize the band. The blots were semi-quantified using gel densitometry with Quantity One software (Bio-Rad, Hercules, CA, USA).

1. **Reverse transcription and semi-quantitative real-time polymerase chain reaction**

Rats were sacrificed by decapitation under deep chloral hydrate (360mg/kg, intraperitoneal) anesthesia. The brain was quickly removed and the lesioned cortex, striatum/corpus callosum of ipsilateral were isolated, snap-frozen on dry ice, and stored at -80°C. Brain samples were homogenized by glass Dounce homogenizer in Trizol (Applied Biosystems, Grand Island, NY, USA). The amount of total mRNA was quantified by ultraviolet spectrophotometry (Beckman, Brea, CA, USA). Reverse transcription was conducted with a RT reagent kit (Promega, Madison, WI, USA) according to manufacturer’s instruction and 1µg RNA was used to synthesize the first strand of cDNA. RT-PCR was performed on Mastercycler realplex analysis system (Eppendorf, Hamburg, Germany), using SYBR green PCR Master Mix (Tiangen Biotech, Beijing, China). Primers were listed in table 1. Relative quantification of target mRNA was normalized to GAPDH of the same sample. The level of the mRNAs was reported as the fold change vs. sham.

**Table 1**. Primers for Real-Time Polymerse Chain Reaction

| Gene | | Primer |
| --- | --- | --- |
| CD86 | SENS: GACACCCACGGGATCAATTA | |
|  | REVS: GCCTCCTCTATTTCAGGTTCAC | |
| CD11b | SENS: ATCCGTAAAGTAGTGAGAGAAC | |
|  | REVS: TCTGCCTCAGGAATGACATC | |
| Il-1β | SENS: AATGACCTGTTCTTTGAGGCTGAC | |
|  | REVS: CGAGATGCTGCTGTGAGATTTGAAG | |
| TNF-α | SENS: CAAGGAGGAGAAGTTCCCAA | |
|  | REVS: CTCTGCTTGGTGGTTTGCTA | |
| CD206 | SENS: ACTGCGTGGTGATGAAAGG | |
|  | REVS: TAACCCAGTGGTTGCTCACA | |
| TGF-β | SENS: CCGCAACAACGCAATCTATG | |
|  | REVS: AGCCCTGTATTCCGTCTCCTT | |

**References**

1. Zhang W, Liu J, Hu X, Li P, Leak RK, Gao Y, et al. N-3 polyunsaturated fatty acids reduce neonatal hypoxic/ischemic brain injury by promoting phosphatidylserine formation and akt signaling. *Stroke*. 2015;46:2943-2950

2. Stetler RA, Cao G, Gao Y, Zhang F, Wang S, Weng Z, et al. Hsp27 protects against ischemic brain injury via attenuation of a novel stress-response cascade upstream of mitochondrial cell death signaling. *J Neurosci*. 2008;28:13038-13055

3. Wang J, Shi Y, Zhang L, Zhang F, Hu X, Zhang W, et al. Omega-3 polyunsaturated fatty acids enhance cerebral angiogenesis and provide long-term protection after stroke. *Neurobiol Dis*. 2014;68:91-103

**Table 2**. List of primary antibody

| Antibody | Company | Cat# | Application | Dilution |
| --- | --- | --- | --- | --- |
| rabbit anti-MBP | Abcam, Cambridge, UK) | ab40390 | IF, WB | 1:500 |
| mouse anti-SMI32 | EMD Millipore, Burlington, MA, USA | NE1023 | IF | 1:1000 |
| mouse anti-APP | EMD Millipore, Burlington, MA, USA | MAB348 | IF | 1:250 |
| rabbit anti-Iba1 | Wako, Tokyo, Japan | 019-19741 | IF | 1:500 |
| rabbit anti-APC | Abcam, Cambridge, UK | ab72040 | IF | 1:250 |
| rabbit anti-NG2 | EMD Millipore, Burlington, MA, USA | 05-710 | IF | 1:250 |
| rabbit anti-CNPase | EMD Millipore, Burlington, MA, USA | MAB326R | WB | 1:500 |
| mouse anti-BrdU | Abcam, Cambridge, UK | ab8152 | IF | 1:250 |

**Supplementary figures**

**S Figure 1**

**
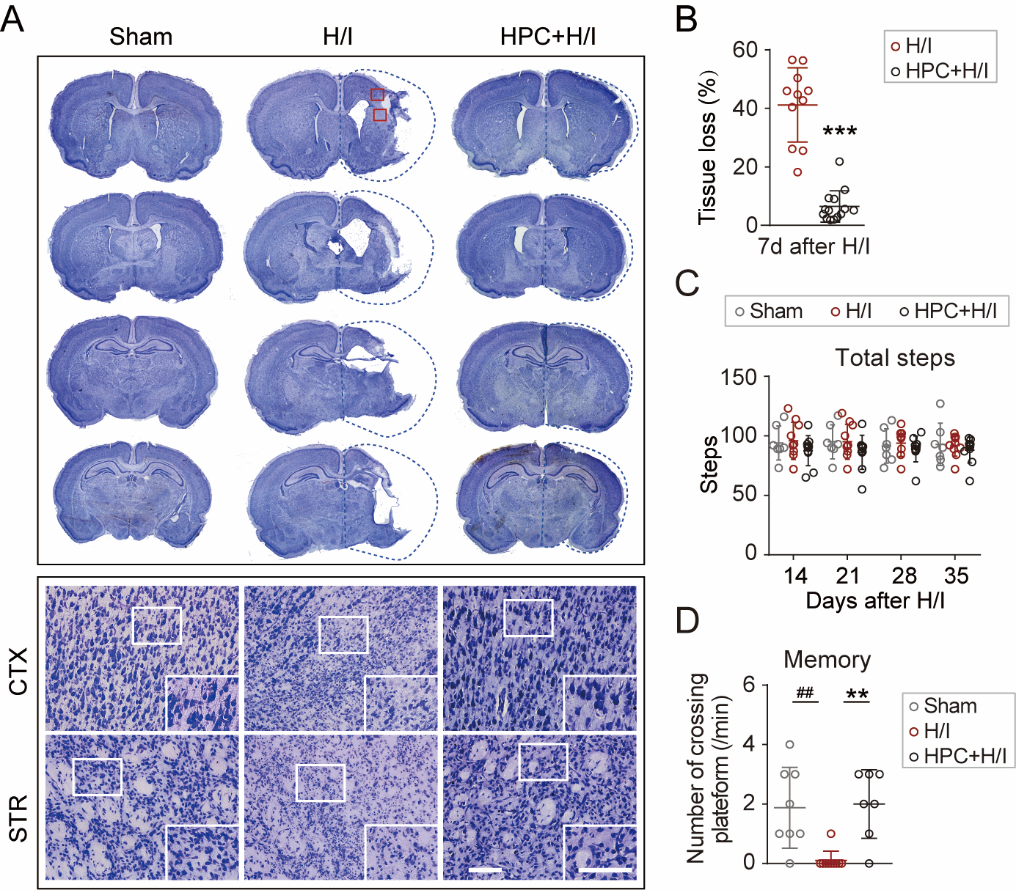
**

**S Figure 1. HPC alleviates H/I-induced tissue loss and long-term neurological deficits**. **(A)** Representative images of cresyl violet-stained brain coronal sections at 7 days after H/I. Dashed lines outline the relative area of the uninjured contralateral hemisphere to depict the tissue loss in ipsilateral hemisphere (up panel). Below are representative high-power images of regions depicted in white rectangles in cortex (CTX) and striatum (STR). The enlarged areas depicting the neuronal cell morphology. Scale bar=100 μm. **(B)** Quantification of tissue loss at 7 days after H/I. Data was expressed percentage of contralateral hemisphere. n=11 rats for H/I, n=14 rats for HPC+H/I. ****p<0.001*, *vs.* H/I group by two-tailed *Student’s* t-test. **(C)** Quantification of total steps in foot fault test at indicated time points after H/I injury. **(D)** Spatial memory was recorded at 35 days after H/I injury by measuring number of crossing the target quadrant. n=7-10 rats per group*. ##p<0.01, vs.* Sham group, ***p<0.01, vs.* H/I group using one-way followed by Bonferroni *post hoc* test.

**S Figure 2**

**
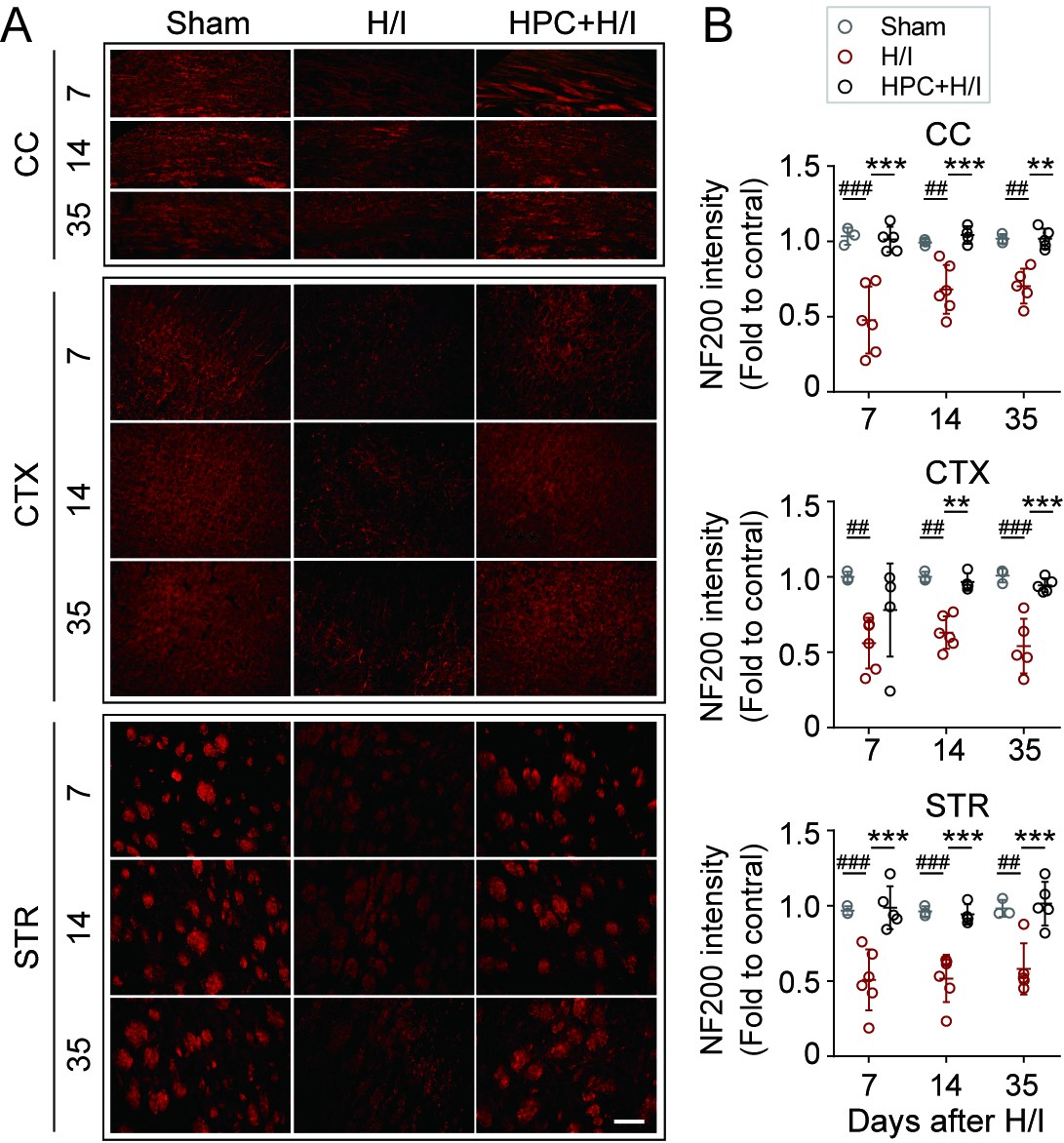
**

**S Figure 2. HPC preserved** **integrity of neurofilament in developing brain after H/I injury**. **(A)** Immunofluorescence labeled staining of NF200 in CC, CTX and STR in ipsilateral hemisphere at 7, 14 and 35 days after H/I injury. Scale bar=100 μm. **(B)** Quantification of the NF200 intensity in ipsilateral hemisphere normalized to contralateral side at the corresponding areas at 7, 14 and 35 days after H/I. n=3-7 rats per group. *#p<0.05, ##p<0.01, ###p<0.001* *vs.* Sham group, **p<0.05*, ***p<0.01, ***p<0.001*, *vs.* H/I group, analyzed using one-way ANOVA followed by Bonferroni *post hoc* test. All data are presented as mean ± SD.

**S Figure 3**


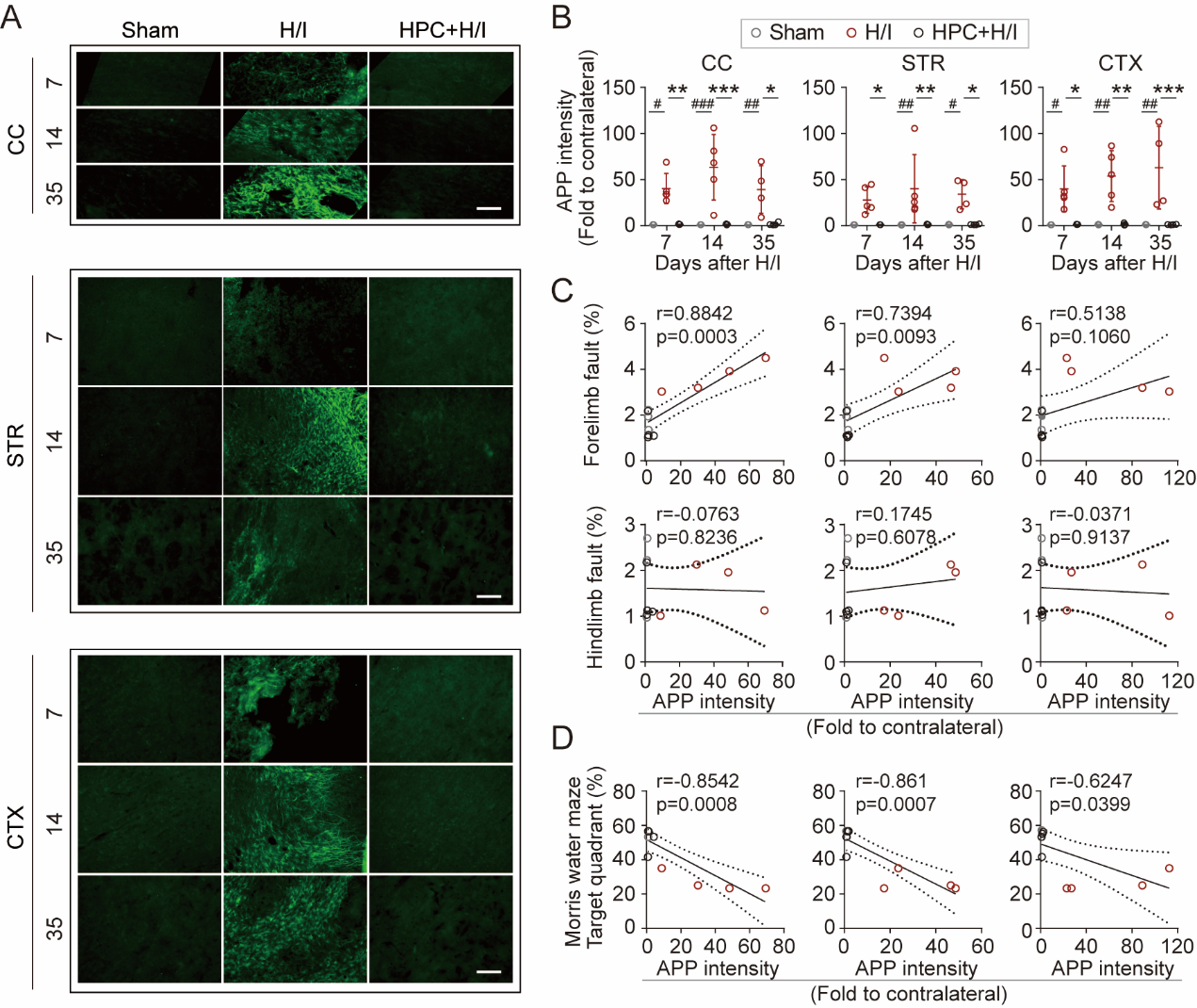


**S Figure 3. HPC alleviates axon damage after H/I injury. (A)** Immunofluorescence staining of APP (green) in CC, STR and CTX in ipsilateral hemisphere at 7, 14 and 35 days after H/I injury. Scale bar=100 μm. **(B)** Quantification of the relative ratio of APP intensity (fold to contralateral hemisphere) in CC, STR and CTX at 7, 14 and 35 days after H/I injury. *#p<0.05, ##p<0.01, ###p<0.001* *vs.* Sham group, **p<0.05*, ***p<0.01, ***p<0.001*, *vs.* H/I group using one-way ANOVA followed by Bonferroni *post hoc* test. Pearson product linear regression analysis between APP intensity in CC (left column), STR (middle column) or CTX (right column) at 35 days after H/I with forelimb fault percentage and hindlimb fault percentage **(C)** in foot fault test or spatial memory (the percentage of time spent in the target quadrant) in Morris water maze **(D)**. n=3-4 rats per group. All data are presented as mean ± SD.

**S Figure 4**


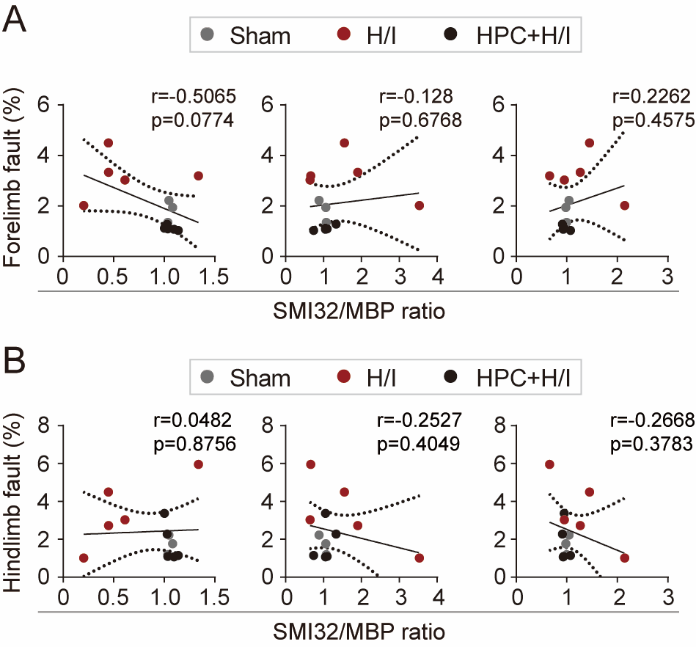


**S Figure 4. Correlation between SMI32/MBP ratio and foot fault percentage in foot fault test.** Correlation of SMI32/MBP ratio in the indicated region (CC, STR and CTX) with forelimb fault percentage **(A)** and hindlimb fault percentage **(B)** in foot fault test. n=3-5 rats for each group.
